# Genomic Insights into the Phosphatidylinositol-Specific Phospholipase C Gene Family in *Leishmania major* and *Leishmania infantum*: Expression Patterns and Potential Association with Drug Resistance

**DOI:** 10.3390/diagnostics15111433

**Published:** 2025-06-05

**Authors:** Serhat Sirekbasan, Samatar Samaleh Osman, Tuğba Gürkök-Tan

**Affiliations:** 1 Department of Medical Laboratory Techniques, Şabanözü Vocational School, Çankırı Karatekin University, 18650 Çankırı, Turkey; serhats@karatekin.edu.tr; 2 Department of Biology, Graduate School of Natural and Applied Sciences, Çankırı Karatekin University, 18100 Çankırı, Turkey; 3 Department of Field Crops, Food and Agriculture Vocational School, Çankırı Karatekin University, 18100 Çankırı, Turkey

**Keywords:** leishmaniasis, genome-wide analysis, antimony resistance, gene expression, clinical diagnostics, molecular markers

## Abstract

**Background/Objectives**: Timely and effective clinical management of leishmaniasis depends on a deep understanding of parasite biology and drug resistance mechanisms. Phosphatidylinositol-specific phospholipase C (PI-PLC) enzymes are critical for parasite survival and immune evasion and possibly influence treatment outcomes. This study aimed to characterize the PI-PLC gene family in the *Leishmania infantum* and *Leishmania major* genomes, with a focus on their expression profiles in antimony-susceptible and -resistant strains to uncover their diagnostic and prognostic relevance. **Methods**: This study conducted a comprehensive genome-wide screening to identify PI-PLC genes in *L. infantum* and *L. major*, followed by detailed analyses of their gene structures, conserved motifs, chromosomal localization, and phylogenetic relationships. To explore potential roles in drug resistance and clinical prognosis, RNA-seq data from antimony-resistant and -susceptible *L. infantum* strains were analyzed for differential gene expression. **Results**: Twenty-two PI-PLC genes were identified in each species, displaying conserved catalytic domains and diverse biochemical characteristics. Phylogenetic and chromosomal analyses revealed gene clustering and distribution patterns. Importantly, expression profiling highlighted several PI-PLC genes with differential regulation in resistant strains, suggesting a role in treatment response and potential as molecular markers. **Conclusions**: Our findings suggest that PI-PLC genes may be associated with drug susceptibility in *L. infantum*, warranting further functional investigation to validate their role as potential molecular markers.

## 1. Introduction

Leishmaniasis is an infectious disease caused by the obligate intracellular parasite *Leishmania* protozoan, transmitted by the bite of its vector, infected sandflies (*Phlebotomus* or *Lutzomyia* spp.) [1]. Globally, approximately 12 million people are estimated to be affected, while an estimated 350 million people are reported to be at risk of infection [2,3]. Although the clinical manifestations caused by the parasite can appear in various forms, they typically encompass a broad spectrum, ranging from skin lesions (cutaneous form) that often result in disfigurement and heal with scarring if left untreated, to potentially fatal visceral involvement (visceral form). The proliferation of *Leishmania* parasites within immune cells, including neutrophils, monocytes, and macrophages, and the subsequent initiation of the immune response against these parasites, constitute the underlying causes of the clinical symptoms observed in the disease [4].

*Leishmania major* and *Leishmania infantum* are two common species that frequently cause disease in humans and can lead to significant health complications. Cutaneous infections are commonly associated with species such as *L. tropica* and *L. major*, while visceral manifestations typically involve species like *L. donovani* and *L. infantum*. Studies have suggested that variations in gene expression regulation may be responsible for the clinical differences observed between *Leishmania* species [5,6]. Due to the absence of an effective vaccine and the resistance of the parasite to current medications, an enhanced understanding of *Leishmania* cell biology is crucial to developing effective treatments. In this context, a deeper exploration of the molecular structures of these species holds vital importance for devising future therapeutic and preventive strategies.

One of the most important processes in the intracellular proliferation of parasites is membrane biogenesis [7]. The production and homeostasis of membranes constitute a complex metabolic network that also involves phospholipid modification and recycling. Phospholipids are degraded by phospholipases, which are classified into four groups A, B, C, and D based on the enzymes’ activities in hydrolyzing specific ester bonds [8]. The enzyme phosphatidylinositol-specific phospholipase C (PI-PLC) plays a vital role in the survival, proliferation, and disease development of many parasitic pathogens, including *Leishmania*. PI-PLC is known to facilitate parasite invasion into host cells, modulate the host cell immune response, and increase the virulence of the pathogen [8,9,10]. Therefore, investigating the role of the PI-PLC enzyme in *Leishmania* species and performing genome-wide characterization of its gene family could offer significant insights into the biology and virulence of these parasites. Furthermore, the inhibition or targeting of this enzyme could be considered as a potential therapeutic strategy to prevent parasite invasion into host cells and alter the disease’s course.

*L. major* was the first species to have its genome sequenced and has been the model for subsequent genome studies of other *Leishmania* species [11]. In 2007, the whole genome sequence of *L. infantum* was sequenced [12]. Although the genome sequences of these parasites are available, the genes involved in various metabolic pathways have not been investigated in detail. In this study, we conducted a comparative study to bioinformatically identify and analyze the PI-PLC gene family in the *L. major* and *L. infantum* genomes.

## 2. Materials and Methods

### 2.1. Identification of PI-PLC Gene Family Members in the L. infantum and L. major Genomes

In this study, genome-wide identification of PI-PLC genes in *L. infantum* and *L. major* parasites was carried out using bioinformatic tools. The genomes of *L. infantum* JPCM5 (assembly ASM287v2) and *L. major* Friedlin strain (assembly ASM272v2) and the PI-PLC sequences of *L. infantum* and *L. major* were downloaded from the NCBI (National Center for Biotechnology Information). The *L. infantum* JPCM5 strain genome was used for analysis because it represents the reference genome for this species and offers high-quality, extensively annotated genomic data, which facilitates reliable comparative and functional genomics [13]. Hidden Markov Model (HMM) profiles for PLC-X (PF00388), PLC-Y (PF00387), and PLC-C2 (PF00168) were retrieved from the Pfam database (http://pfam.xfam.org/ [accessed on 8 May 2024]) and used to identify genes using HMMER Version 3.0 (http://hmmer.janelia.org/ [accessed on 3 July 2024]) with default parameters (E-value < e^−20^). The NCBI CDD (http://www.ncbi.nlm.nih.gov/cdd/ [accessed on 3 July 2024]) database was used to validate all candidate proteins. The InterPro and Pfam databases were employed to confirm sequence verification. Biochemical properties such as isoelectric point (pI), molecular weight (MW), and protein size were identified using the online ExPASy tool (https://web.expasy.org/compute_pi/ [accessed on 15 July 2024]). The subcellular distribution of proteins were predicted using CELLO Version 2.5 (http://cello.life.nctu.edu.tw/ [accessed on 23 July 2024]).

### 2.2. Chromosome Locations and Synteny Analysis

For chromosomal localization of the LiPLCs and LmPLCs, the start and end points of the genes were retrieved from NCBI. Genetic maps were drawn using the online tool MapGene2Chrom Version 2 (http://mg2c.iask.in/mg2c_v2.1/index_cn.html [accessed on 25 July 2024]).

### 2.3. Conserved Motif and Gene Structure Analysis

The TBtools software Version 1.12 was used to visualize the *L. infantum* and *L. major* LiPLCs and LmPLCs gene structures [14]. The online software MEME Suite Version 5.5.5 (https://meme-suite.org/meme/tools/meme [accessed on 25 July 2024]) was used to perform motif prediction analysis of the *L. infantum* and *L. major* PI-PLCs [15].

### 2.4. Gene Expression Profiling and Functional Analysis

The identified LiPI-PLC genes were used for detecting the gene expression data from RNA-seq data available in the SRA database (https://www.ncbi.nlm.nih.gov/sra [accessed on 30 July 2024]) under project number PRJNA348689 [16]. RNA-seq data from resistant (SRR5574933 and SRR5575024) and susceptible (SRR5575022, SRR5575025, SRR5575026) *L. infantum* lines to potassium antimonyl tartrate (Sb^III^) were used for expression analysis. The clean reads were aligned to the *L. infantum* JPCM5 reference genome by using the HISAT2 algorithm. DESeq2 was utilized to identify differentially expressed genes, with genes having an adjusted *p*-value < 0.05 and fold-change > 1.5 considered as differentially expressed. Gene Ontology enrichment analysis was conducted using AmiGO Version 2 (http://amigo.geneontology.org/ [accessed on 30 July 2024]) [17].

## 3. Results

### 3.1. The Bioinformatic Identification of PI-PLCs in L. infantum and L. major

In this study, the HMM tool Version 3 was used to screen the conserved domains PLC-C, PLC-X, and PLC-Y in the genomes of *L. infantum* and *L. major*. A total of 22 PI-PLC gene family members were identified in *L. infantum* and *L. major*, individually. The basic physicochemical properties and gene characteristics are presented in Table 1 for *L. infantum* and Table 2 for *L. major*. The analyses revealed that PI-PLC family members contained at least one domain in *Leishmania* species, with the PLC-C domain being the most abundant. The coding sequence (CDS) lengths of the 22 LiPLC genes ranged from 738 bp (XP_001467341.1) to 7029 bp (XP_001462688.1), and the CDS lengths of the 22 LmPLC genes ranged from 804 (XP_001685053.1) to 6726 (XP_003721595.1). Both LiPI-PLC and LmPI-PLC genes consisted of only one exon without introns (Supplementary Figures S1 and S2).

We also performed the physicochemical characterization of all PI-PLC protein sequences. The protein lengths for the LiPI-PLCs ranged from 245 amino acids (aa) (XP_001467341.1) to 2342 aa (XP_001462688.1), while the LmPI-PLC proteins ranged from 267 aa (XP_001685053.1) to 2241 aa (XP_003721595.1). The highest molecular weight for the LiPI-PLCs was 251.464 kDa, and for the LmPI-PLCs it was 241.67 kDa. The average molecular weights were 101.102 kDa for *L. infantum* and 107.364 kDa for *L. major*. The isoelectric point (pI) for the LiPI-PLCs ranged from 4.40 (XP_001464332.1) to 9.46 (XP_001464095.2) with a median of 7.32, while for the LmPI-PLCs, the pI ranged from 4.40 (XP_001687745.1) to 9.41 (XP_001681820.1) with a median of 7.28. Subcellular localization predictions, based on in silico analysis using CELLO, suggest that the proteins are primarily located in the nucleus, plasma membrane, and cytoplasm; however, these localizations have not been experimentally validated.

### 3.2. Phylogenetic and Conserved Motif Analysis of the LiPI-PLC and LmPI-PLC Proteins

**Figure 1:**
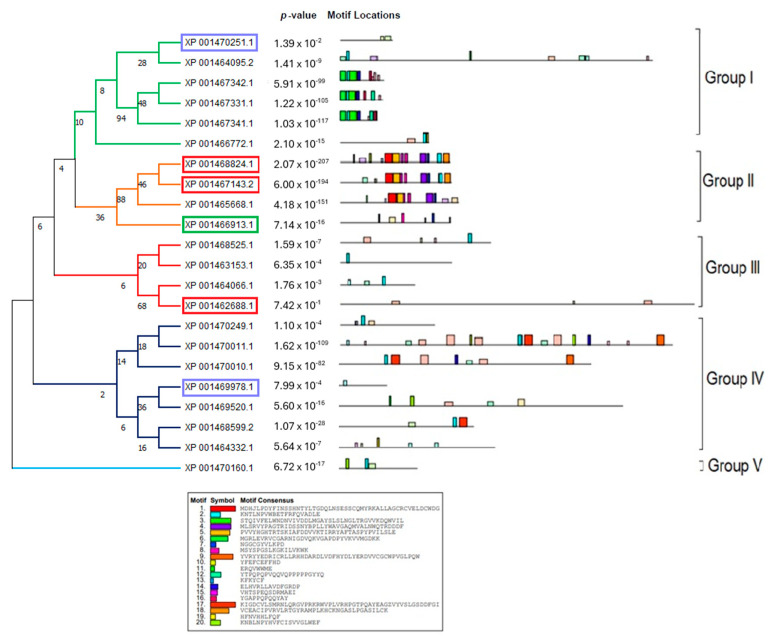
Phylogenetic analysis of the LiPI-PLC gene family in *L. infantum*. The tree was constructed using protein sequences of the PI-PLC gene family, highlighting classification into five groups.

**Figure 2:**
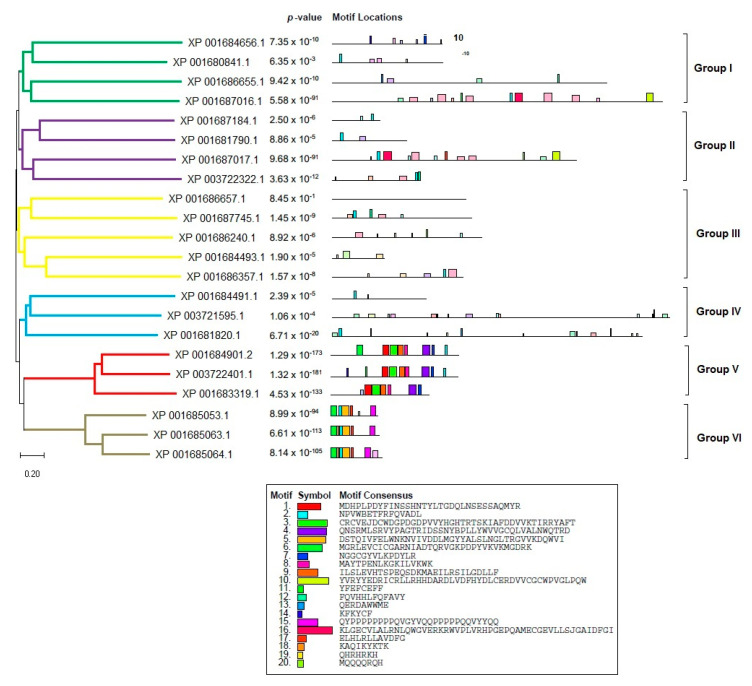
Phylogenetic analysis of the LmPI-PLC gene family in *L. major*. The tree illustrates six distinct groups based on protein sequences.

In order to accurately depict the evolutionary relationships of the LiPI-PLC and LmPI-PLC proteins, we conducted phylogenetic analyses. Since there were not enough sequences in other species, we performed analyses within species and between *L. infantum* and *L. major*. Based on protein sequences, the LiPI-PLC gene family was classified into five groups, with Group IV consisting of the highest number of proteins, while Group V had only one representative (Figure 1). In *L. major*, a total of 22 LmPI-PLCs were classified into six groups, with Group III containing the highest number of proteins (Figure 2).

The concatenated motif structures of the PI-PLC protein sequences we identified in *Leishmania* species were also analyzed using MEME Suite (Supplementary Figure S3). In particular, the comparative motif profiles of the LmXP_001685053.1 and LiXP_001467331.1 proteins show that these two proteins are highly similar and are likely orthologous genes. Sequence alignment revealed 87% identity among these genes, suggesting they may have arisen from recent gene duplication events.

**Figure 3:**
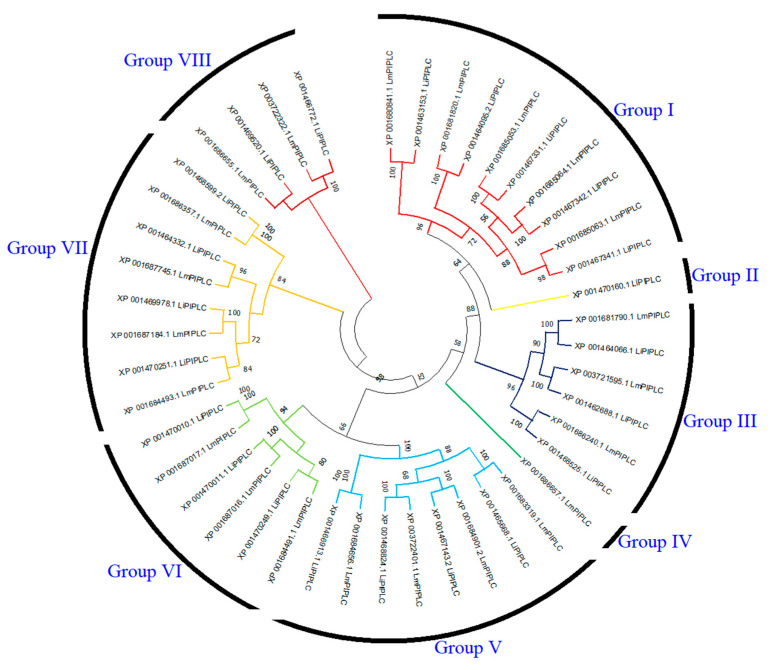
Comparative phylogenetic tree of PI-PLC proteins in *L. infantum* and *L. major*. PI-PLC proteins were grouped into eight subfamilies based on sequence homology and evolutionary divergence. Different colors represent different subfamilies.

The phylogenetic tree of PI-PLC genes based on protein sequences from *L. infantum* and *L. major* is shown in Figure 3. In the phylogenetic tree, PI-PLC family proteins were grouped into eight subfamilies. LiPI-PLC XP001470160.1 and LmPI-PLC XP001686657.1 were clustered separately. Group I had the highest number of PI-PLC family members.

### 3.3. Chromosome Distribution, Gene Structure, and Synteny Analysis of the LiPI-PLC and LmPI-PLC Genes

The chromosomal localization was conducted using MG2C. According to the gene locus data, 22 LiPI-PLC and LmPI-PLC genes were mapped to 12 chromosomes (Chr01, Chr06, Chr13, Chr14, Chr22, Chr28, Chr29, Chr30, Chr31, Chr34, Chr35, and Chr36). Chr36 had the highest number of genes in both species (Supplementary Figures S4 and S5). To understand the evolutionary relationships of the LiPI-PLC and LmPI-PLC genes, syntenic analysis was performed between them. The results showed that the LiPI-PLC and LmPI-PLC genes were closely related.

### 3.4. Expression Pattern of the Genes

**Figure 4:**
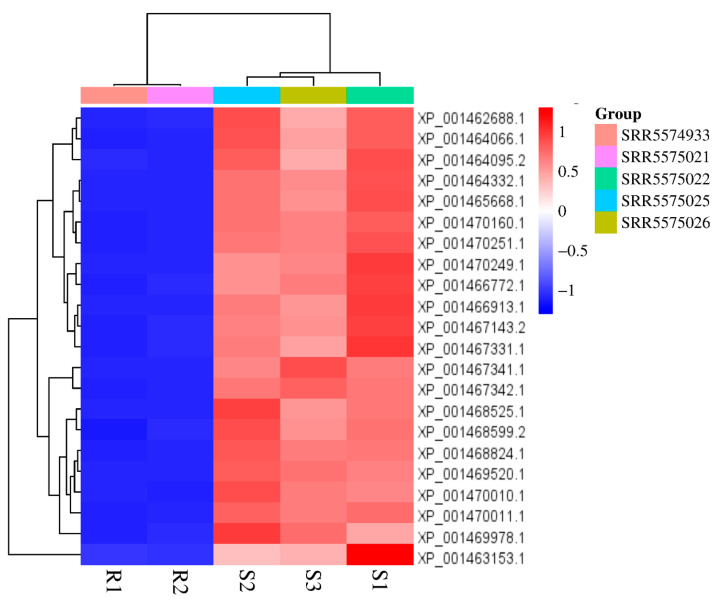
Heatmap of PI-PLC gene expression profiles in susceptible and resistant *L. infantum* lines. The heatmap illustrates the differential expression of the PI-PLC genes based on RNA-seq data.

The transcriptomic profiles showed PI-PLC expression in *L. infantum*. The analysis revealed that PI-PLC genes were expressed at higher levels in susceptible lines of *L. infantum* (Figure 4). Out of the 22 LiPI-PLC transcripts, four of them were significantly expressed. All the transcripts, XP_001468599.2 (1.65-fold), XP_001469978.1 (2.01-fold), XP_001469520.1 (2.13-fold), and XP_001470011.1 (2.36-fold), were upregulated in the Sb^III^-susceptible *L. infantum*.

### 3.5. Gene Ontology

**Figure 5:**
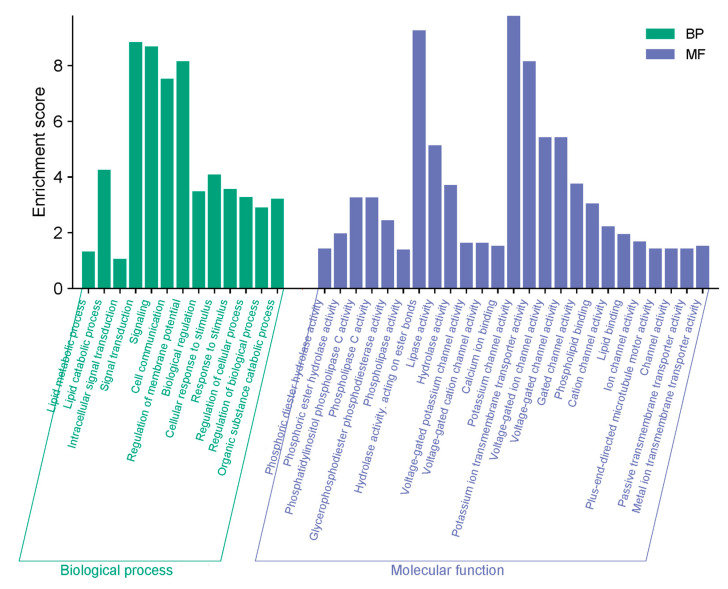
Predicted gene ontology analysis of the PI-PLC genes in *L. infantum* and *L. major*. The GO enrichment analysis identifies the most significant biological processes and molecular functions associated with the PI-PLC genes.

In the Gene Ontology (GO) and KEGG analysis of the PI-PLC genes, only biological process (BP) and molecular function (MF) information were accessible. We identified 13 items under BP and 25 items under MF. The most enriched BP items included signal transduction, signaling, and regulation of membrane potential. Under MF, potassium channel activity, hydrolase activity acting on ester bonds, and potassium ion transmembrane transporter activity were significantly enriched (Figure 5).

## 4. Discussion

Leishmaniasis is a major global health concern caused by protozoan parasites of the genus *Leishmania*. Present in over 100 countries, it manifests in various clinical forms, most notably cutaneous and visceral leishmaniasis. Despite the existence of treatment options, increasing drug resistance continues to drive the search for novel therapeutic strategies [18].

Phospholipases are known to modulate host immune responses by generating lipid mediators that either promote inflammation or suppress immune functions. For example, deletion of the phospholipase C gene in *Toxoplasma gondii* reduced parasite replication and virulence in mice [19], while inhibition of phospholipase A2 in *Plasmodium falciparum* led to diminished parasite growth and increased susceptibility to antimalarials [20]. These examples underscore the therapeutic potential of targeting phospholipase genes.

In this study, we performed a genome-wide characterization of the PI-PLC gene family in *L. infantum* and *L. major*. To our knowledge, this is the first detailed genomic investigation of PI-PLCs in *Leishmania* species. We identified 22 genes containing PI-PLC-X, PI-PLC-Y, and C2 domains. Phylogenetic analysis revealed that LiPI-PLC and LmPI-PLC members clustered into five and six distinct groups, respectively. All but two proteins (LiXP_001470160.1 and LmXP_001686657.1) formed species-specific clusters, indicating high conservation.

To provide preliminary insights into the potential functions of the *Leishmania* PLC proteins identified in this study, we inferred their subcellular localization and key physicochemical features. In the context of *Leishmania* biology, subcellular localization is a critical factor. Membrane-associated or secreted PLCs may participate in host–parasite interactions, signaling cascades, or immune evasion mechanisms [21,22]. Therefore, predicting the localization of PLC proteins based on their physicochemical characteristics facilitates the development of testable hypotheses regarding their roles in parasite virulence and survival. These predictions form the foundation for future functional studies, including experimental validation through proteomics or localization assays. The biochemical properties and chromosomal localization of clustered PI-PLC proteins were nearly identical across both species, suggesting functional conservation and evolutionary stability. However, the two divergent genes, LiXP_001470160.1 and LmXP_001686657.1, differed significantly in sequence characteristics, localization, and potentially, function. Notably, LiXP_001470160.1 was localized to the plasma membrane, while its *L. major* counterpart was nuclear, implying that these genes may play species-specific roles in host–parasite interactions.

In particular, the high level of similarity between the motif profiles of LmXP_001685053.1 and Li XP_001467331.1 suggests that they may be orthologous genes. The presence of similar motif clusters in the N-terminal regions of both proteins indicates functionally conserved catalytic regions. In particular, the presence of motif sequences in the same positions supports the evolutionary derivation of these genes from a common ancestor. This indicates the functional importance of the PI-PLC family and also reveals the conserved structure of the genes across species. This highlights the evolutionary stability of the PI-PLC gene family in *Leishmania* species and suggests that these genes may play critical roles in host–pathogen interactions.

In the light of motif and sequence similarity analyses, it was evaluated that XP_001685053.1 and LiXP_001467331.1 genes, apart from interspecies orthologs, may have been formed by genomic duplication events in the past and may have been preserved in multi-copy regions, especially on chromosome 31. It is known that chromosome 31 is frequently supernumerary in *Leishmania* genomes and hosts many virulence factors. Therefore, the functional role as well as the evolutionary origin of this gene pair is worth investigating in further studies.

Given that PI-PLC proteins are known to be involved in signal transduction, immune modulation, and membrane remodeling, their conserved nature suggests a possible role in parasite survival and virulence. Interestingly, although the PI-PLC gene family is structurally conserved between *L. major* and *L. infantum*, the distinct clinical manifestations of these species suggest that species-specific regulation and localization of the PI-PLCs may contribute to pathogenic differences. These variations could influence clinical outcomes such as disease progression and treatment response, highlighting a potential role for PI-PLC genes in clinical prognosis and phenotype-based diagnosis [23,24].

Furthermore, previous studies have shown that gene expression profiles are strongly linked to antimony resistance in *Leishmania*. Resistant strains often display upregulation of stress response and resistance-related genes and downregulation of genes such as mitogen-activated protein kinase 1 (MAPK1) and aquaglyceroporin (AQP), both of which are implicated in drug uptake and efflux mechanisms [25,26,27].

Both *L. major* and *L. infantum* were found to possess 22 PI-PLC family members, indicating a high level of conservation in gene count between the two species. This conservation suggests that the PI-PLC repertoire may be essential for core biological processes in *Leishmania* spp. Despite having the same number of PI-PLC genes, subtle sequence variations or divergence in regulatory elements may lead to species-specific expression profiles and functional adaptations.

When compared to the known PI-PLC families in other eukaryotes, the *Leishmania* PI-PLCs exhibit partial structural similarity, such as the presence of conserved catalytic X and Y domains. However, the typical regulatory domains found in mammalian PLC families are largely absent or highly divergent. This suggests that these parasite enzymes may not fit neatly into classical PI-PLC families [28,29]. We propose that *Leishmania* PI-PLC family members may constitute a divergent and functionally specialized set of proteins adapted to parasitic life.

The genes LmXP_001685053.1 and LiXP_001467331.1, members of the PI-PLC family, are notable for their highly conserved motif structures and similar expression profiles. Such genes represent promising candidates as potential biomarkers and therapeutic targets for both diagnosis and treatment. Their conserved features suggest potential utility in RNA-based diagnostics, development of specific inhibitors, resistance prediction, and patient management strategies. However, further validation through in vitro and in vivo studies—such as gene knockout/knockdown experiments and pharmacological inhibition assays—will be essential to confirm their functional relevance.

## 5. Conclusions

In our study, we observed that the PI-PLC gene family was significantly upregulated in antimony-susceptible *L. infantum* strains compared to resistant strains. This suggests that PI-PLC genes may be downregulated as part of an adaptive resistance mechanism, potentially affecting antimony uptake or immune evasion strategies. These results underscore the potential clinical relevance of PI-PLC genes not only as molecular markers for predicting drug susceptibility but also as targets for precision therapy.

Overall, our findings indicate that the PI-PLC gene family may influence key clinical outcomes in leishmaniasis, including disease progression, treatment efficacy, and resistance development. Thus, PI-PLC genes may serve as valuable molecular biomarkers in clinical settings for both diagnostic and prognostic purposes, as well as targets for novel antileishmanial therapies.

## Figures and Tables

**Table 1:** Basic physicochemical properties and gene characteristics of *L. infantum* PI-PLC genes.

NCBI ID	Pfam Domain	Length (aa)	Nucleotide (bp)	MW (kDa)	pI	Gravy	Chr	Predicted Subcellular Location (with Confidence Scores)
XP_001462688.1	PLC-X, PLC-Y, PLC-C2	2342	7029	251.464	8.49	−0.281	1	Nuclear (2.973)
XP_001463153.1	PLC-C2	739	2220	75.202	6.97	−0.017	6	Nuclear (2.943)
XP_001464066.1	PLC-C2	497	1494	55.638	7.87	−0.342	13	Nuclear (2.307)
XP_001464095.2	PLC-C2	2062	6189	217.924	9.46	−0.228	13	Nuclear (3.386)
XP_001464332.1	PLC-C2	1006	3021	111.607	4.40	−0.872	14	Nuclear (2.119) Cytoplasmic (1.848)
XP_001465668.1	PLC-C2	784	2355	87.832	6.67	−0.327	22	Extracellular (1.559)
XP_001470160.1	PLC-C2	508	1527	55.893	6.03	−0.071	28	Plasma Membrane (2.351)
XP_001470249.1	PLC-C2	627	1884	69.034	5.79	−0.034	28	Plasma Membrane (1.478)
XP_001470251.1	PLC-X	349	1050	39.361	9.16	−0.048	28	Plasma Membrane (1.789) Mitochondrial (1.308)
XP_001466772.1	PLC-C2	589	1770	66.12	8.44	−0.321	29	Nuclear (1.658) Mitochondrial (1.398) Cytoplasmic (1.058)
XP_001466913.1	PLC-X, PLC-Y	730	2193	80.02	7.85	−0.254	30	Mitochondrial (1.335)
XP_001467143.2	PLC-X, PLC-Y, PLC-C2	737	2214	82.37	8.06	−0.356	30	Nuclear (2.021)
XP_001467331.1	PLC-C2	282	849	32.054	8.45	−0.627	31	Nuclear (2.978)
XP_001467341.1	PLC-C2	245	738	26.66	5.79	−0.282	31	Nuclear (1.948)
XP_001467342.1	PLC-C2	288	867	31.097	8.35	−0.456	31	Nuclear (2.438)
XP_001468525.1	PLC-C2	995	2988	106.76	6.65	−0.375	34	Nuclear (2.716)
XP_001468599.2	PLC-C2	870	2613	100.047	6.88	−0.884	34	Nuclear (2.265) Cytoplasmic (1.708)
XP_001468824.1	PLC-X, PLC-Y, PLC-C2	729	2190	82.023	7.31	−0.259	35	Extracellular (1.963)
XP_001469520.1	PLC-C2	1827	5484	198.29	6.53	−0.144	36	Nuclear (1.716) Plasma Membrane (1.170)
XP_001469978.1	PLC-X	316	951	35.993	5.60	−0.302	36	Cytoplasmic (2.049)
XP_001470010.1	PLC-C2	1625	4878	177.451	9.12	−0.511	36	Nuclear (2.777)
XP_001470011.1	PLC-C2	2193	6582	241.394	7.15	−0.190	36	Nuclear (2.010) Plasma Membrane (1.522)

**Table 2:** Basic physicochemical properties and gene characteristics of *L. major* PI-PLC genes.

NCBI ID	Pfam Domain	Length (aa)	Nucleotide (bp)	MW (kDa)	pI	Gravy	Chr	Predicted Subcellular Location (with Confidence Scores)
XP_003721595.1	PLC-C2	2241	6726	241.67	8.47	−0.304	Chr1	Nuclear
XP_001680841.1	PLC-C2	737	2214	75.123	6.19	−0.007	Chr6	Nuclear (2.697)
XP_001681790.1	PLC-C2	497	1494	55.616	8.28	−0.338	Chr13	Nuclear (1.979)
XP_001681820.1	PLC-C2	2063	6192	218.31	9.41	−0.243	Chr13	Nuclear (3.563)
XP_001687745.1	PLC-C2	930	2793	103.97	4.40	−0.776	Chr14	Nuclear (1.983) Cytoplasmic (1.770)
XP_001683319.1	PLC-X, PLC-Y, PLC-C2	555	1670	61.904	5.95	−0.312	Chr22	Nuclear (1.196)
XP_001684491.1	PLC-C2	627	1884	68.996	5.88	−0.036	Chr28	Plasma Membrane (1.670)
XP_001684493.1	PLC-X	349	1050	39.225	8.98	−0.073	Chr28	Plasma Membrane (1.843) Mitochondrial (1.447)
XP_003722322.1	PLC-X	589	1770	66.097	8.67	−0.306	Chr29	Nuclear (2.324)
XP_001684656.1	PLC-X, PLC-Y, PLC-C2	730	2193	79.914	7.52	−0.240	Chr30	Mitochondrial (1.264) Cytoplasmic (1.069)
XP_001684901.2	PLC-X, PLC-Y, PLC-C2	737	2214	82.276	8.43	−0.326	Chr30	Nuclear (1.498) Mitochondrial (1.274)
XP_001685053.1	PLC-C2	267	804	30.426	8.11	−0.597	Chr31	Nuclear (2.869)
XP_001685063.1	PLC-C2	274	825	29.92	5.90	−0.154	Chr31	Nuclear (1.839) Extracellular (1.153)
XP_001685064.1	PLC-C2	288	867	31.37	8.76	−0.475	Chr31	Nuclear (2.304)
XP_001686240.1	PLC-C2	995	2988	106.554	6.34	−0.404	Chr34	Nuclear (2.597)
XP_001686357.1	PLC-C2	870	2613	100.203	7.09	−0.893	Chr34	Nuclear (2.218) Cytoplasmic (1.738)
XP_003722401.1	PLC-X, PLC-Y, PLC-C2	729	2190	82.085	7.93	−0.246	Chr35	Extracellular (2.188)
XP_001686655.1	PLC-C2	1827	5484	197.973	6.36	−0.097	Chr36	Nuclear (1.624) Plasma Membrane (1.423)
XP_001686657.1	PLC-C2	888	2667	94.912	6.35	−0.387	Chr36	Nuclear (3.005)
XP_001687016.1	PLC-C2	2193	6582	241.368	6.69	−0.162	Chr36	Nuclear (1.918) Plasma Membrane (1.760)
XP_001687017.1	PLC-C2	1625	4878	177.055	8.99	−0.494	Chr36	Nuclear (2.979)
XP_001687184.1	PLC-C2	316	951	177.055	5.50	−0.253	Chr36	Cytoplasmic (1.949)

## Data Availability

Most of the data generated or analyzed are included in the article. The remaining datasets used and/or analyzed during the current study are available from the corresponding author upon request.

## Supplementary Materials

The following supporting information can be downloaded at: https://www.mdpi.com/article/10.3390/diagnostics15111433/s1, Figure S1: Gene structure analysis of the *L. infantum* PI-PLC genes; Figure S2: *L. major* gene structure; Figure S3: Conserved motifs identified in the PI-PLC proteins from *L. infantum* and *L. major* using combined MEME analysis; Figure S4: Chromosomal distribution of the PI-PLC genes in *L. infantum*; Figure S5: Chromosomal distribution of the PI-PLC genes in *L. major*.

## Abbreviations

The following abbreviations are used in this manuscript:

PI-PLC	phosphatidylinositol-specific phospholipase
NCBI	National Center for Biotechnology Information
HMM	Hidden Markov Model
pI	isoelectric point
MW	molecular weight
SRA	sequence read archive
GO	Gene Ontology
BP	biological process
MF	molecular function
MAPK1	mitogen-activated protein kinase 1
AQP	aquaglyceroporin
