# Supplementary material for: Genomic Insights into the Phosphatidylinositol-Specific Phospholipase C Gene Family in Leishmania major and Leishmania infantum: Expression Patterns and Potential Association with Drug Resistance
Source: Diagnostics (Basel). 2025 Jun 5;15(11):1433. doi: 10.3390/diagnostics15111433 (PMC12155177; doi:10.3390/diagnostics15111433)
Supplement: Supplementary file 1 [file diagnostics-15-01433-s001.zip › diagnostics-3651313-supplementary.pdf]

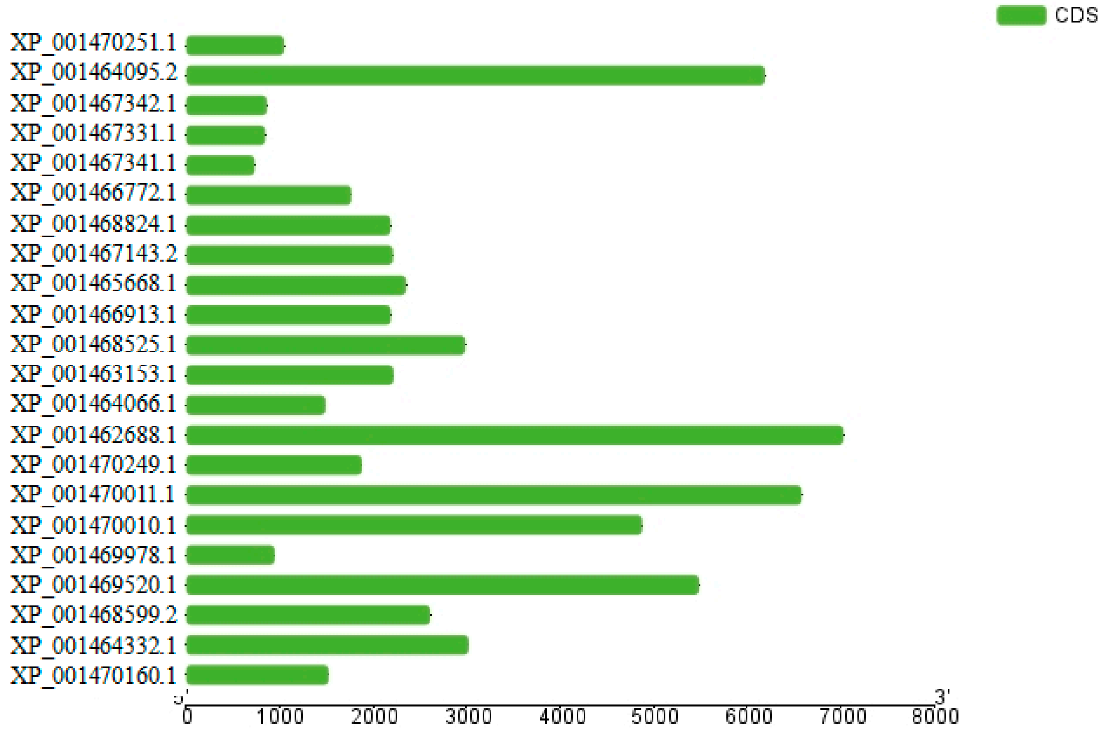

Supplementary Figure S1. Gene structure analysis of *L. infantum* PI-PLC genes. This figure represents the gene structures of PI-PLC family members in *L. infantum*. The analysis highlights exon-intron organization, showing that all genes consist of a single exon without introns.

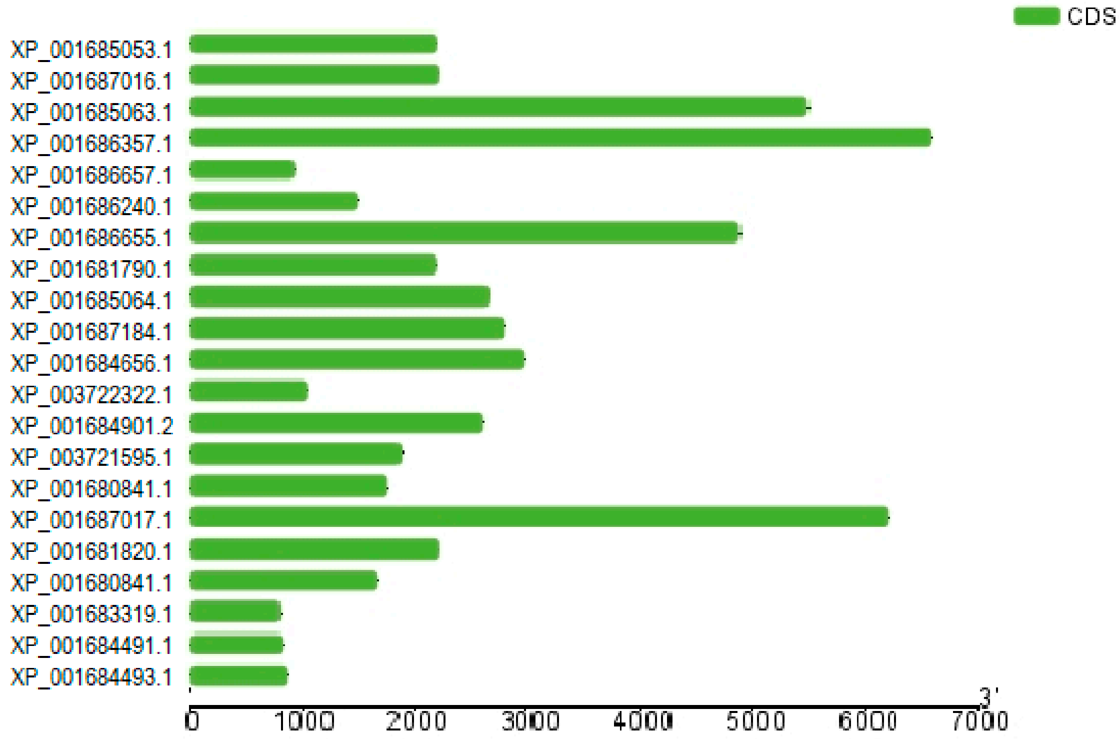

Supplementary Figure S2. *L. major* gene structure. The figure illustrates the gene structures of PI-PLC family members in *L. major*.

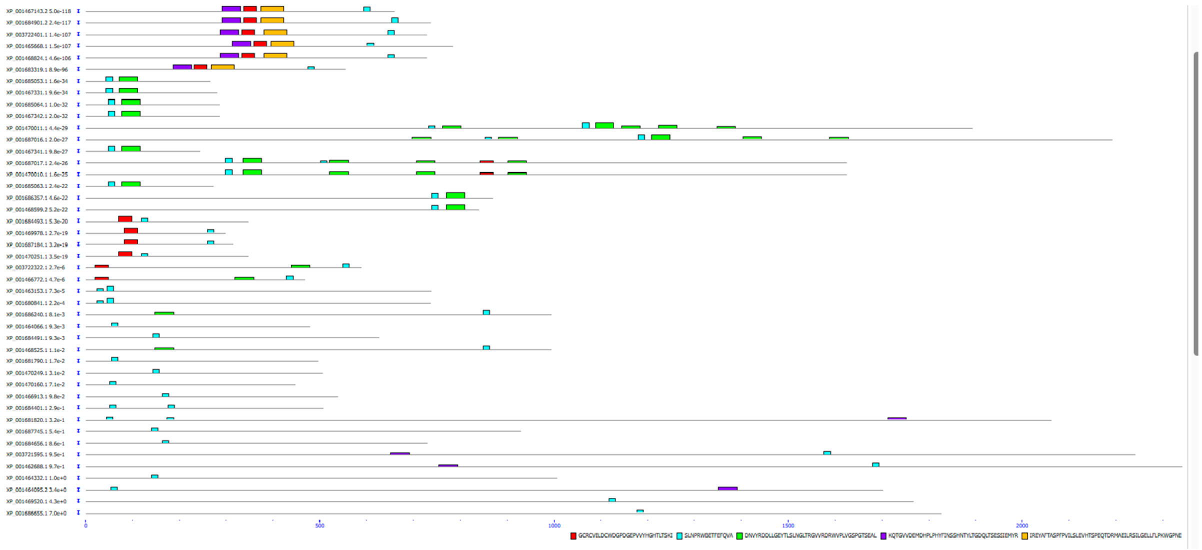

Supplementary Figure S3. Conserved motifs identified in PI-PLC proteins from *L. infantum* and *L. major* using combined MEME analysis.

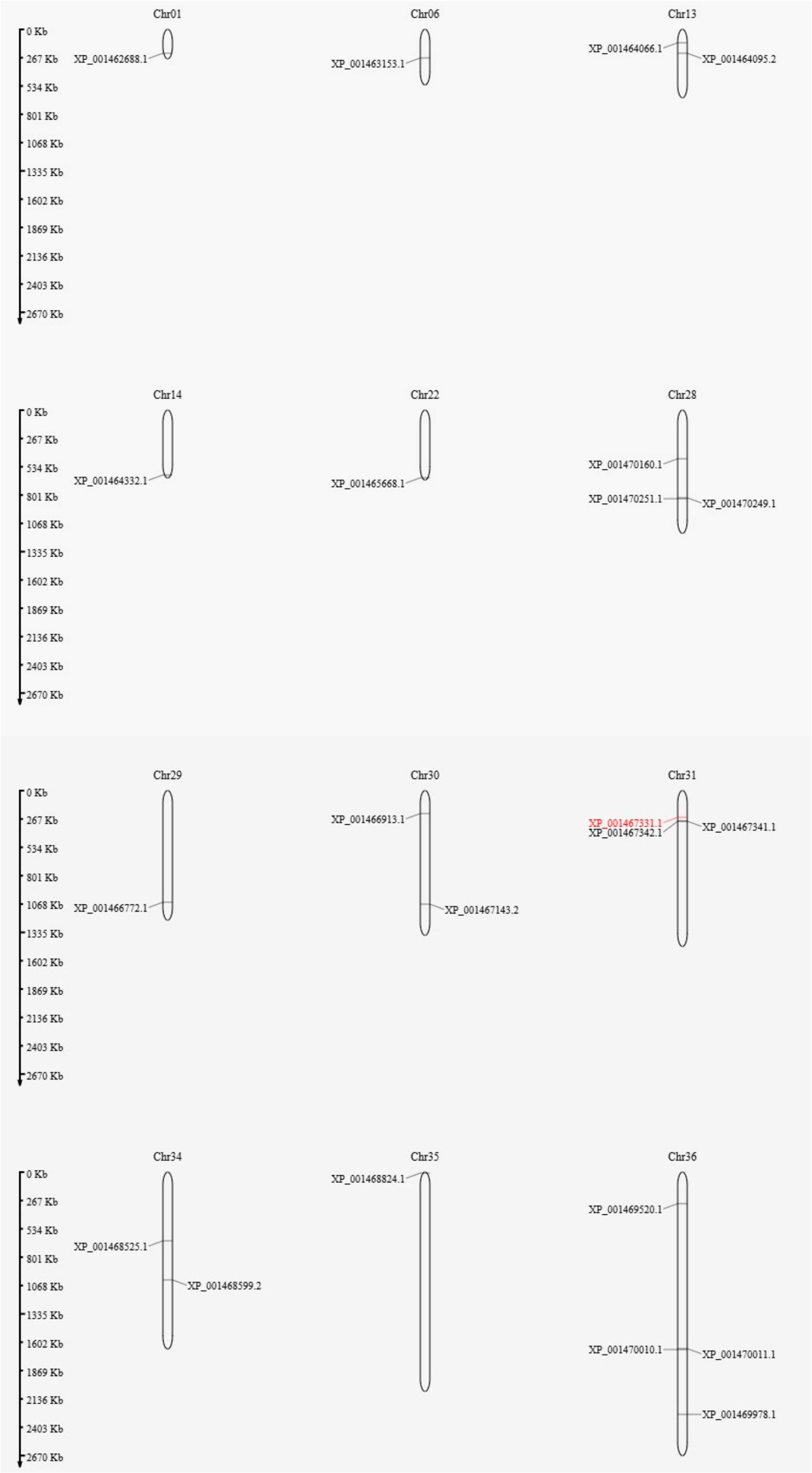

Supplementary Figure S4. Chromosomal distribution of PI-PLC genes in *L. infantum*.

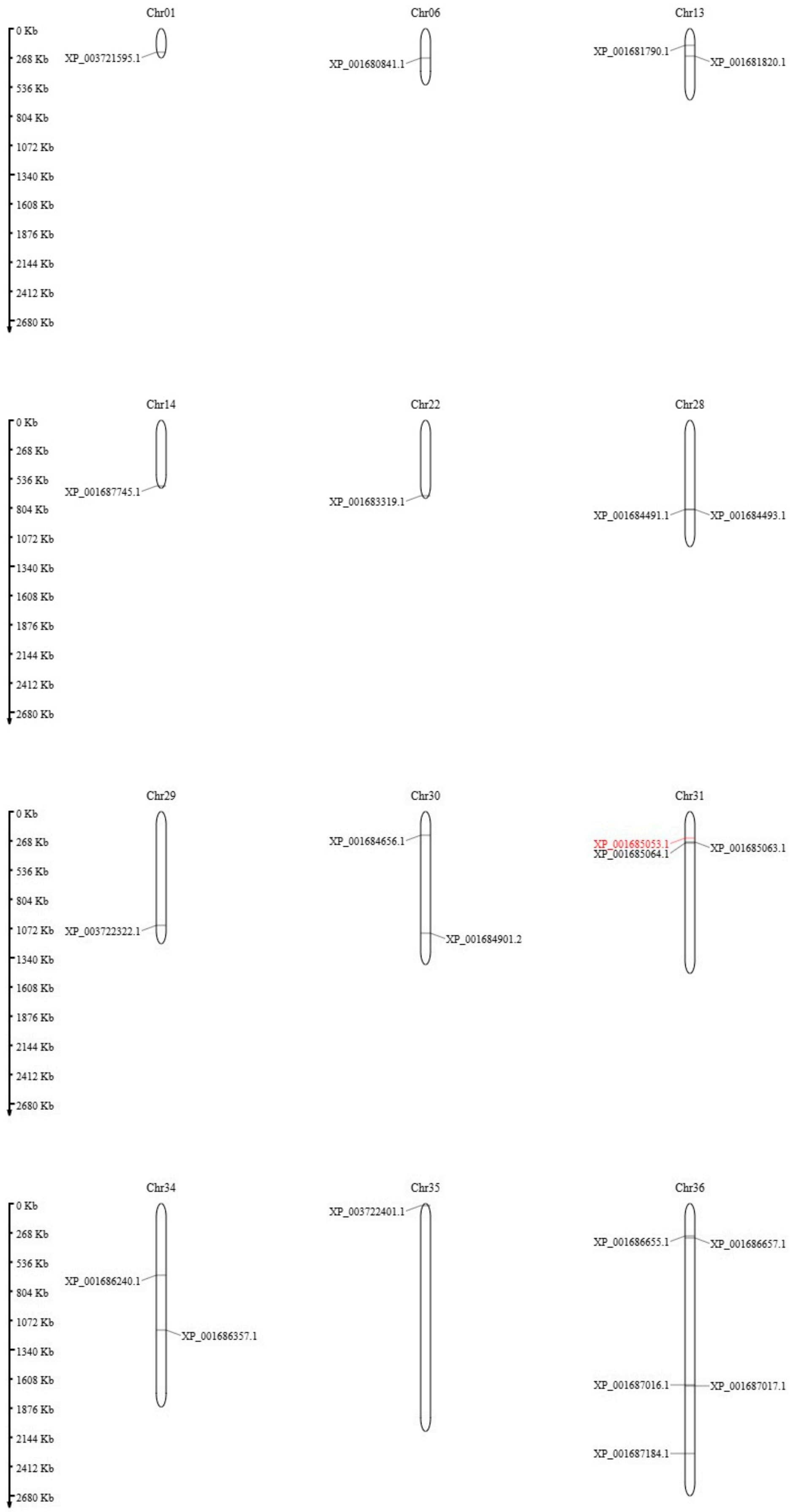

Supplementary Figure S5. Chromosomal distribution of PI-PLC genes in *L. major*.
